# Elucidation of metabolic pathways of 25-hydroxyvitamin D3 mediated by CYP24A1 and CYP3A using *Cyp24a1* knockout rats generated by CRISPR/Cas9 system

**DOI:** 10.1016/j.jbc.2021.100668

**Published:** 2021-04-15

**Authors:** Kaori Yasuda, Miyu Nishikawa, Kairi Okamoto, Kyohei Horibe, Hiroki Mano, Mana Yamaguchi, Risa Okon, Kimie Nakagawa, Naoko Tsugawa, Toshio Okano, Fumihiro Kawagoe, Atsushi Kittaka, Shinichi Ikushiro, Toshiyuki Sakaki

**Affiliations:** 1 Department of Pharmaceutical Engineering, Faculty of Engineering, Toyama Prefectural University, Imizu, Toyama, Japan; 2 Department of Biotechnology, Faculty of Engineering, Toyama Prefectural University, Imizu, Toyama, Japan; 3 Laboratory of Hygienic Sciences, Faculty of Pharmaceutical Sciences, Kobe Gakuin University, Chuo-ku, Kobe, Japan; 4 Department of Health and Nutrition, Faculty of Health and Nutrition, Osaka Shoin Women's University, Higashi-Osaka, Japan; 5 Department of Hygienic Sciences, Kobe Pharmaceutical University, Higashinada-ku, Kobe, Japan; 6 Faculty of Pharmaceutical Sciences, Teikyo University, Tokyo, Japan

**Keywords:** vitamin D, metabolism, cytochrome P450, CYP24A1, CRISPR/Cas9, 1,25(OH)_2_D3, 1α,25-dihydroxyvitamin D_3_, 25(OH)D3, 25-hydroxyvitamin D_3_, ADX, adrenodoxin, ADR, adrenodoxin reductase, PTH, parathyroid hormone, VDR, vitamin D receptor

## Abstract

CYP24A1-deficient (*Cyp24a1* KO) rats were generated using the CRISPER/Cas9 system to investigate CYP24A1-dependent or -independent metabolism of 25(OH)D3, the prohormone of calcitriol. Plasma 25(OH)D3 concentrations in *Cyp24a1* KO rats were approximately twofold higher than in wild-type rats. Wild-type rats showed five metabolites of 25(OH)D3 in plasma following oral administration of 25(OH)D3, and these metabolites were not detected in *Cyp24a1* KO rats. Among these metabolites, 25(OH)D3-26,23-lactone was identified as the second major metabolite with a significantly higher T_max_ value than others. When 23*S*,25(OH)_2_D3 was administered to *Cyp24a1* KO rats, neither 23,25,26(OH)_3_D3 nor 25(OH)D3-26,23-lactone was observed. However, when 23*S*,25*R*,26(OH)_3_D3 was administered to *Cyp24a1* KO rats, plasma 25(OH)D3-26,23-lactone was detected. These results suggested that CYP24A1 is responsible for the conversion of 25(OH)D3 to 23,25,26(OH)_3_D3 *via* 23,25(OH)_2_D3, but enzyme(s) other than CYP24A1 may be involved in the conversion of 23,25,26(OH)_3_D3 to 25(OH)D3-26,23-lactone. Enzymatic studies using recombinant human CYP species and the inhibitory effects of ketoconazole suggested that CYP3A plays an essential role in the conversion of 23,25,26(OH)_3_D3 into 25(OH)D3-26,23-lactone in both rats and humans. Taken together, our data indicate that *Cyp24a1* KO rats are valuable for metabolic studies of vitamin D and its analogs. In addition, long-term administration of 25(OH)D3 to *Cyp24a1* KO rats at 110 μg/kg body weight/day resulted in significant weight loss and ectopic calcification. Thus, *Cyp24a1* KO rats could represent an important model for studying renal diseases originating from CYP24A1 dysfunction.

Vitamin D_3_ is metabolized by CYP2R1 and CYP27A1 to 25-hydroxyvitamin D_3_ (25(OH)D3) in the liver and by CYP27B1 to 1α,25-dihydroxyvitamin D3 (1,25(OH)_2_D3) in the kidneys, thereby binding to vitamin D receptors (VDRs) and exhibiting various physiological effects. The key enzyme in the metabolism of 25(OH)D3 and 1,25(OH)_2_D3 is CYP24A1, which is known to generate multiple metabolites *via* C-24 and C-23 hydroxylation pathways (1, 2).

There are two vitamin D responsive elements in the promoter region of the *CYP24A1* gene, and the binding of active vitamin D to VDRs leads to profound transcriptional induction of CYP24A1. The resultant CYP24A1 protein inactivates the active form of vitamin D. This mechanism is crucial for maintaining constant plasma levels of active vitamin D. CYP24A1 dysfunction causes elevated plasma levels of 1,25(OH)_2_D3 and is associated with idiopathic infantile hypercalcemia or kidney stones, which indicates the importance of this enzyme for vitamin D metabolism (3, 4).

We expressed rat and human CYP24A1 in *Escherichia coli* and examined their enzymatic properties (5, 6). We identified that rat CYP24A1 catalyzes a six-step reaction commencing with 24*R*-position hydroxylation, leading to calcitroic acid, which is designated as the C-24 pathway. In contrast, human CYP24A1 catalyzes a four-step reaction starting at position 23*S*, leading to the formation of a 26,23-lactone.

The ratios of the C-23 and C-24 pathways vary among species. For human CYP24A1, the ratio in the initial reactions of C-23 and C-24 is approximately 1:4, whereas in rat CYP24A1, it is approximately 1:25 (6, 7), based on the comparison of first step metabolic reaction. In species such as guinea pig and opossum, the C-23 pathway is, in contrast, predominant (7, 8).

However, when 25(OH)D3 was administered to wild-type (WT) rats and mice, 25(OH)D3-26,23-lactone, which is the final product of 25(OH)D3 in the C-23 pathway, was produced as a major metabolite (9, 10, 11). This fact strongly suggests that enzymes other than CYP24A1 are involved in the C-23 pathway in rats and mice. To date, Masuda *et al.* (12) administered tritium-labeled 25(OH)D3 to hetero- and *Cyp24a1* KO mice and analyzed their metabolites in the plasma, liver, kidneys, and small intestine. Their results showed that metabolites of 25(OH)D3, 24,25(OH)_2_D3, 24-oxo-25(OH)D3, and 24-oxo-23,25(OH)_2_D3 were abundant in plasma of hetero-mice, but none were detected in *Cyp24a1* KO mice, indicating the importance of CYP24A1 in the metabolism of 25(OH)D3 (12).

Recently, it has become possible to quantify various vitamin D metabolites more sensitively using LC/MS/MS, and Kaufmann *et al.* have succeeded in quantifying various metabolites by performing analysis after derivatization by DMEQ-TAD (13, 14, 15, 16). They demonstrated that metabolites in the C-24 and C-23 pathways, such as 24-oxo-25(OH)D3 (C-24), 24-oxo-23,25(OH)_2_D3 (C-24), and 25(OH)D3-26,23-lactone (C-23), are not detected in *Cyp24a1* KO mice (13), suggesting that Cyp24A1 plays a crucial role in both C-23 and C-24 pathways in mice. Our focus is investigating which steps of the C-24 and C-23 pathways are carried out only by CYP24A1 and which steps contribute to the extent of involvement by other enzymes. Although *Cyp24a1* KO mice appear to be useful, it is difficult to evaluate detailed time courses of metabolites in terms of blood sampling volumes possible in small rodents.

Recently, we successfully generated genetically modified (GM) rats deficient in *Cyp27b1* or *Vdr* genes (17). Type II rickets model rats with a mutant *Vdr* (R270L), which recognizes 1,25(OH)_2_D3 with an affinity equivalent to that of 25(OH)D3, were also generated. Although *Cyp27b1*-knockout (KO), *Vdr-*KO, and *Vdr* (R270L) rats exhibit rickets symptoms, they are significantly different to each other. Comparison among WT and three types of GM rats led to novel concepts regarding vitamin D actions, including genomic and nongenomic actions.

In this study, we generated *Cyp24a1* KO rats and succeeded in measuring the time course of 25(OH)D3 plasma levels and its metabolites after the administration of 25(OH)D3. Furthermore, we revealed for the first time that not only CYP24A1, but also CYP3A1/2 is involved in the bioconversion of 23*S*,25,26(OH)_3_D3 to 25(OH)D3-26,23-lactone, which is known to have a biological activity to reduce serum Ca level (18).

## Results

### Comparison of 25(OH)D3 metabolism between an *in vitro* system containing rat recombinant CYP24A1 and an *in vivo* system using WT rats

**Figure 1:**
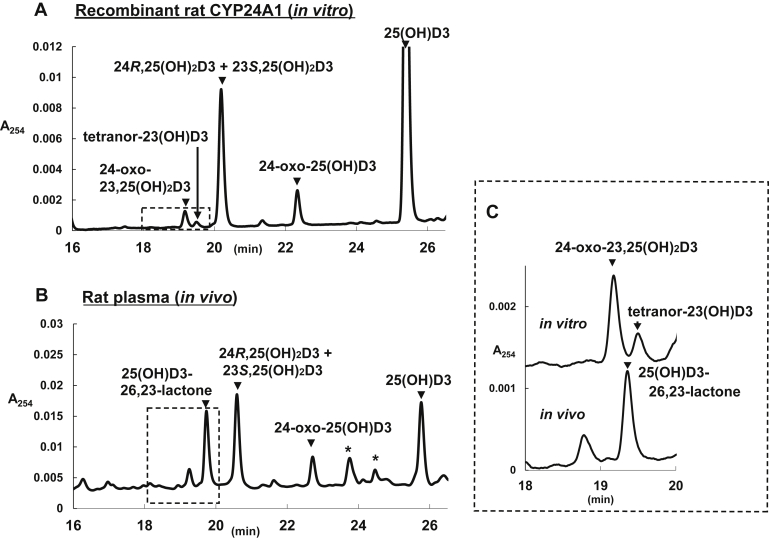
**HPLC profiles of 25(OH)D3 and its metabolites *in vitro* (*A*) and *in vivo* (*B*).***A*, indicates the metabolites by recombinant rat CYP24A1 and (*B*) indicates the metabolites of plasma of wild-type rats administrated 25(OH)D3. *C*, is the comparison of the metabolites at retention time of 18.0–20.0 min between (*A*) and (*B*). Peaks with *arrow* indicate the metabolites of 25(OH)D3, whereas peaks labeled mark (∗) are not the metabolites-derived peaks.

In order to obtain authentic standards for 25(OH)D3 metabolites, 25(OH)D3 was metabolized in a reconstituted system containing rat recombinant CYP24A1 expressed in *E. coli* cells. As shown in Figure 1*A*, 24*R*,25(OH)_2_D3, 23*S*,25(OH)_2_D3, 24-oxo-25(OH)D3, 24-oxo-23,25(OH)_2_D3, and tetranor-23(OH)D3 were isolated as authentic standards. Although 24*R*,25(OH)_2_D3 and 23*S*,25(OH)_2_D3 were eluted at the same retention time (Fig. 1*A*), they were clearly separated by normal-phase HPLC, as described previously (6). All of the metabolites standards showed an absorbance maximum at 265 nm. The purity of 24*R*,25(OH)_2_D3, 24-oxo-25(OH)D3, 24-oxo-23,25(OH)_2_D3, and tetranor-23(OH)D3 was estimated to be 94, 92, 84, 95%, respectively, based on HPLC-UV profiles.

Figure 1*B* presents the HPLC profile of 25(OH)D3 and its metabolites in the plasma of WT rats 24 h after 25(OH)D3 administration. A metabolite at a retention time of 19.8 min, which was not detected *in vitro*, was detected in rat plasma (Fig. 1, *B* and *C*). The DMEQ-TAD adduct of this metabolite showed the same LC retention time and similar MS and MS/MS patterns to those of the synthetic standard of (23*S*,25R)-25(OH)D3-26,23-lactone. These results appear to be consistent with the finding that one of the major metabolites of 25(OH)D3 in the plasma of 25(OH)D3-treated rats is (23*S*,25*R*)-25(OH)D3-26,23-lactone (9, 10, 19). Thus, the peak observed at a retention time of 19.8 min (Fig. 1*B*) was identified as (23*S*,25*R*)-25(OH)D3-26,23-lactone.

### Simultaneous quantification of 25(OH)D3 and its metabolites

Ion spectra involving DMEQ-TAD adducts of 25(OH)D3, 24,25(OH)_2_D3, and 25(OH)D3-26,23-lactone were similar to those shown in our previous study (11). The ion product spectra of DMEQ-TAD adducts of 24,25(OH)_2_D3, 24-oxo-25(OH)D3, 24-oxo-23,25(OH)_2_D3, tetranor-23(OH)D3, 25(OH)D3-26,23-lactone, and 23,25,26(OH)_3_D3 are shown in Figure S1. The major characteristic ions for DMEQ-TAD adducts of 24,25(OH)_2_D3, 24-oxo-25(OH)D3, 24-oxo-23,25(OH)_2_D3, tetranor-25(OH)D3, 25(OH)D3-26,23-lactone, and 23,25,26(OH)_3_D3 were their molecular ion species [M + H] at m/z of 762.4, 760.4, 776.4, 690.4, 774.4, and 778.4, respectively, and fragment ions at m/z 247 and 468 were detected as major ion products of all compounds (Fig. S1). The ion fragment at m/z 468 was selected for multiple reaction monitoring (MRM) analysis, as shown in Table S1, because of greater specificity and lower background than the fragment at m/z 247. Chromatograms for each metabolite are shown in Figure S1. Two peaks for each metabolite were detected, which represented the 6*R*- and 6*S*- isomers of the DMEQ-TAD adduct of each metabolite, as in the case of 25(OH)D3 reported in a previous study (20). The mixtures of each authentic standard could be quantitatively analyzed simultaneously using MRM scan mode.

### Generation of *CYP24a1* KO rats

Twenty-six offspring were obtained, one of which had a 1 bp insertion at a target site. Consequently, 30 amino acid residues from 439 to 469 were substituted, resulting in the introduction of a premature stop codon at residue 469 (Fig. S2). This frame shift causes loss of the cysteine residue at position 462, which is the fifth ligand of heme iron and the active center, and causes a deletion involving the C-terminal portion of CYP24A1, which consists of 514 amino acids. Thus, this truncated CYP24A1 could not show enzymatic activity. Homozygotes for this mutant CYP24A1 allele were used in this study. We confirmed that no off-target site (OTS) events occurred in potential OTSs searched using the CRISPR Direct tool for CYP24A1 sgRNA (Fig. S3).

### Appearance, growth, and bone-metabolism-related parameters in the blood of *Cyp24a1* KO rats

**Figure 2:**
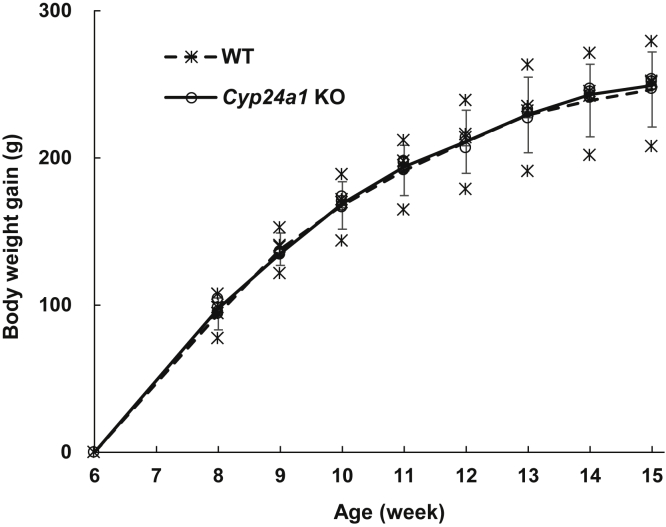
**Comparison of growth between WT and *Cyp24a1* KO rats.** The *dotted* and *solid lines* indicate the average of the individual data of WT (*asterisk*) and *Cyp24a1* KO (*open circle*) rats, respectively (n = 4; biological replicates). Error bars represent standard deviation.

No significant difference was observed in the appearance and growth rate between *Cyp24a1* KO and WT rats (Fig. 2). Femoral length in *Cyp24a1* KO rats at 15 weeks of age was similar to that of WT rats. CT scanning, von Kossa staining, and toluidine blue staining of femora revealed no noticeable differences between *Cyp24a1* KO and WT rats (Fig. S4).

St-Arnaud (21) demonstrated that 50% of *Cyp24a1* KO mice (homozygous mutant) died before 3 weeks of age, but no such situation was observed with *Cyp24a1* KO rats. μCT scans showed no particular symptoms such as soft tissue calcification (data not shown). Neither plasma Ca and P levels nor parathyroid hormone (PTH) levels were significantly different between *Cyp24a1* KO and WT rats (Table 1).

**Table 1:** Plasma concentration of Ca, P, and PTH of WT and *Cyp24a1* KO rats at 15 weeks of age

Plasma Ca, P, and PTH levels	WT	CYP*24a1* KO
Ca (mg/dl)	9.4 ± 0.4	9.5 ± 0.5
P (mg/dl)	7.9 ± 0.6	7.5 ± 0.2
PTH (pg/ml)	200 ± 90	260 ± 29

Each value represents the mean ± SD (n = 5–8; biological replicates).

### Comparison of plasma concentrations of 25(OH)D3 and its metabolites in *Cyp24a1* KO and WT rats

**Figure 3:**
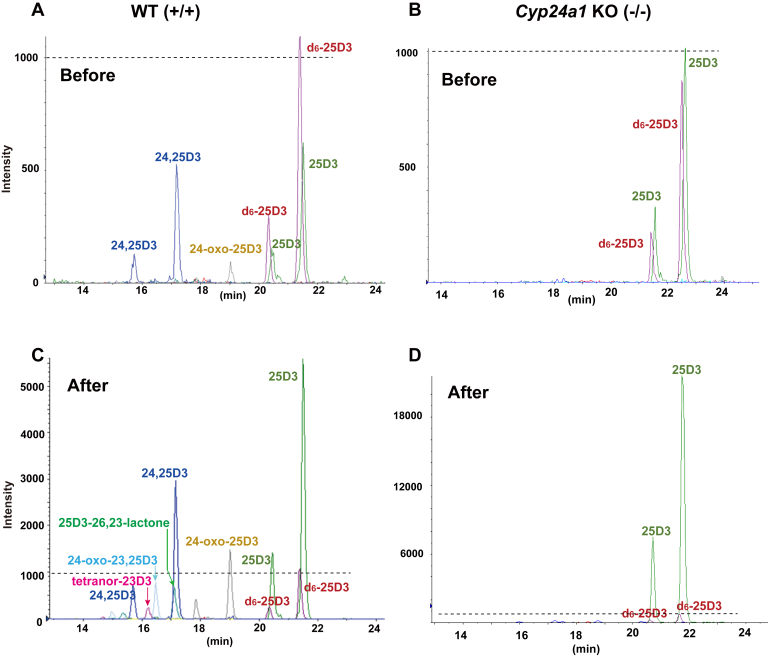
**MRMchromatograms of DMEQ-TAD adducts of 25(OH)D3 and its metabolites in the plasma of WT (*****A*****and*****C*****) and*****Cyp24a1*****KO (*****B*****and*****D*****) rats before (*****A*****and*****B*****) and 4** **h after (*****C*****and*****D*****) single administration of 25(OH)D3.***Dotted lines* indicate the same intensity.

Plasma concentrations of 25(OH)D3 and its metabolites in WT and *Cyp24a1* KO rats before 25(OH)D3 administration were compared (Fig. 3, *A* and *B* and Table 2). 25(OH)D3, 24,25(OH)_2_D3, and 24-oxo-25(OH)D3 were detected in the plasma of WT rats. As shown in Table 3, approximately twice as much 25(OH)D3 was detected in the plasma of *Cyp24a1* KO rats than in the plasma of WT animals, whereas 24,25(OH)_2_D3 and 24-oxo-25(OH)D3 were not detected (<0.8 nM and 1 nM for 24,25(OH)_2_D3 and 24-oxo-25(OH)D3, respectively), which suggested that CYP24A1-dependent metabolites were not detected in *Cyp24a1* KO rats. Plasma concentrations of 25(OH)D3, 24,25(OH)_2_D3, and 24-oxo-25(OH)D3 in *Cyp24a1* mutant-heterozygous rats were 15.4 ± 8.1, 22.0 ± 10.2, and 5.6 ± 2.1 nM, respectively, similar to those of WT rats. Plasma concentrations of 1,25(OH)_2_D3 in WT and *Cyp24a1* KO rats were 29 ± 14 pg/ml (70 ± 33 pM) and 21 ± 7 pg/ml (49 ± 16 pM), respectively, and no significant difference was observed between them (Table 2).

**Table 2:** Plasma concentration of 25(OH)D3 and its metabolites of WT and *Cyp24a1* rats at 15 weeks of age

Vitamin D3 metabolites	WT	*Cyp24a1* KO
	nM	nM
25(OH)D3	20.0 ± 7.1	44.3 ± 3.4
24,25(OH)_2_D3	17.8 ± 6.9	n.d.
24-oxo-25(OH)D3	3.9 ± 1.9	n.d.
1,25(OH)_2_D3	0.070 ± 0.033	0.049 ± 0.016

Each value represents the mean ± SD (n = 5–7; biological replicates).

n.d. indicates less than 0.8 nM and 1 nM for 24,25(OH)_2_D3 and 24-oxo-25(OH)D3, respectively.

**Table 3:** T_max_ and C_max_ values of 25(OH)D3 and its each metabolite with single dose administration of 25(OH)D3 to WT or *Cyp24a1* KO rats at 15 weeks of age

Strain	Vitamin D3 metabolites	T_max_ (h)	C_max_ (nM)
WT	25(OH)D3	3.0 ± 1.1	370 ± 150
	24,25(OH)_2_D3	4.3 ± 0.8	436 ± 164
	24-oxo-25(OH)D3	4.7 ± 1.0	101 ± 29
	24-oxo-23,25(OH)_2_D3	4.8 ± 1.8	84 ± 31
	tetranor-23(OH)D3	5.0 ± 2.0	6.8 ± 1.3
	26,23-lactone-25(OH)D3	10.7 ± 2.1	195 ± 48
*Cyp24a1* KO	25(OH)D3	5.3 ± 1.0	1460 ± 630

The values are shown as the mean ± SD. (n = 5–7; biological replicates).

**Figure 4:**
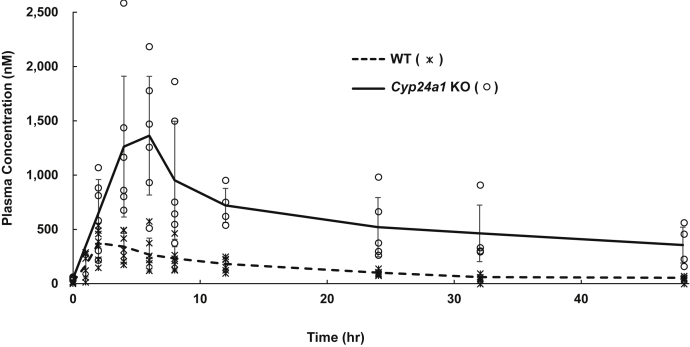
**Time courses of plasma 25(OH)D3 concentration in WT and *Cyp24a1* KO rats after single dose administration of 25(OH)D3.** The *dotted* and *solid lines* indicate the average of WT (*asterisk*) and *Cyp24a1* KO (*open circle*) rats, respectively (n = 5–7; biological replicates). Error bars represent standard deviation.

**Figure 5:**
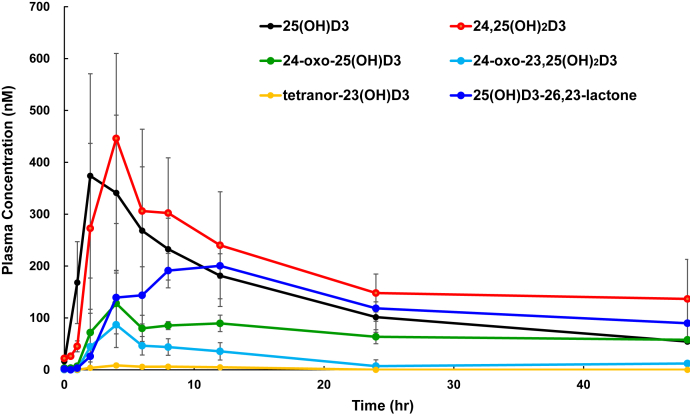
**Time courses of plasma concentration of 25(OH)D3 and its metabolites in WT rats after single dose administration of 25(OH)D3.** The values are shown as the mean ± SD (n = 5–7; biological replicates).

In the next step, 25(OH)D3 was orally administered to WT or *Cyp24a1* KO rats at 200 μg/kg body weight, and plasma concentrations of 25(OH)D3 and its metabolites were examined periodically over 0–48 h. After 25(OH)D3 administration, plasma 25(OH)D3 concentrations in *Cyp24a1* KO rats were markedly elevated than in WT animals (Figs. 3 and 4). The maximum plasma concentration (C_max_) of 25(OH)D3 in *Cyp24a1* KO rats was four times higher than that in WT rats (Table 3 and Fig. 4). Figure 3, *C* and *D* show the MRM chart of 25(OH)D3 and its metabolites in rat plasma 4 h after administration of 25(OH)D3. In addition to 24,25(OH)_2_D3 and 24-oxo-25(OH)D3, three metabolites 24-oxo-23,25(OH)_2_D3, tetranor-23(OH)D3, and 25(OH)D3-26,23-lactone were detected in WT rats, whereas none of these were detected in *Cyp24a1* KO rats. Figure 5 shows the time course of plasma concentration of all metabolites detected in WT rats. Among the five metabolites detected in WT rats, 24,25(OH)_2_D3, 24-oxo-25(OH)D3, and 25(OH)D3-26,23-lactone remained at high plasma concentrations 48 h after administration. It is noted that they are known to have a high vitamin D binding protein (DBP)-binding affinity (22). It is reasonable to assume that the half-life (t_1/2_) in plasma of these metabolites is closely linked to their affinity for DBP.

Among the five metabolites, other than 25(OH)D3-26,23-lactone, the time to maximum plasma level (T_max_) was approximately 4 h (4.3 ± 0.8, 4.7 ± 1.0, 4.8 ± 1.8, 5.0 ± 2.0 h for 24,25(OH)_2_D3, 24-oxo-25(OH)D3, 24-oxo-23,25(OH)_2_D3, and tetranor-25(OH)D3, respectively), whereas only 25(OH)D3-26,23-lactone exhibited markedly higher values (10.7 ± 2.1 h; Table 3). Previous studies demonstrated that 25(OH)D3-26,23-lactone is one of the major metabolites in rats and mice upon 25(OH)D3 administration (9, 10), which agrees with our present results. The T_max_ values of 25(OH)D3-26,23-lactone compared with those of other metabolites, with no detection of 25(OH)D3-26,23-lactone in our *in vitro* system containing recombinant rat CYP24A1 (Fig. 1), strongly suggested the likely involvement of enzyme(s) other than CYP24A1 in the formation of 25(OH)D3-26,23-lactone.

### Identification of enzymes involved in 25(OH)D3-26,23-lactone formation

**Figure 6:**
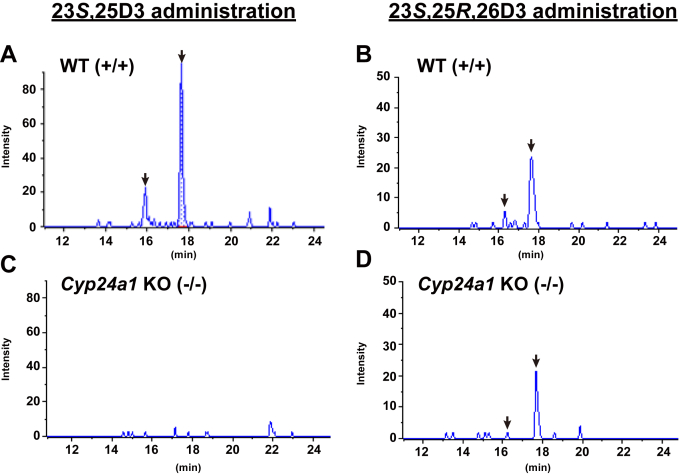
**MRM chromatograms of DMEQ-adducts of 26,23-lactone-25(OH)D3 (m/z 774.4→468.1) in the plasma of WT (*****A*****and*****B*****) and*****Cyp24a1*****KO (*****C*****and*****D*****) rats 6** **h after single administration of 23*****S*****,25(OH)**_**2**_**D3 (*****A*****and*****C*****) and 23*****S*****,25*****R*****,26(OH)**_**3**_**D3 (*****B*****and*****D*****)**. *Arrows* indicate the peaks derived 26,23-lactone-25(OH)D3.

**Figure 7:**
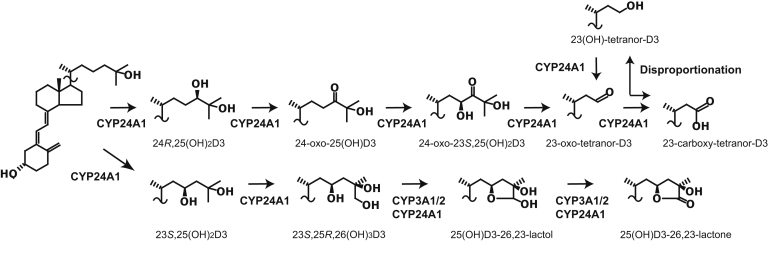
**25(OH)D3 metabolic pathway in rats based on the previous** (2, 35) **and current studies**.

As described above, 25(OH)D3-26,23-lactone has been previously reported to be detected as a 25(OH)D3 metabolite, and its structure has been reported to be (23*S*,25*R*)-26,23-lactone-25(OH)D3(19). It has also been reported that this metabolite is generated from 25(OH)D3 *via* 23*S*,25(OH)_2_D3 and 23*S*,25*R*,26(OH)_3_D3 (9, 23). To investigate the participation of CYP24A1 and other enzyme(s) in 25(OH)D3-26,23-lactone formation *in vivo*, 23*S*,25(OH)_2_D3 and 23*S*,25*R*,26(OH)_3_D3 were separately administered to *Cyp24a1* KO rats and examined for the formation of 25(OH)D3-26,23-lactone. As shown in Figure 3, no conversion of 25(OH)D3 into 23*S*,25(OH)_2_D3 was observed in *Cyp24a1* KO rats. When 23*S*,25(OH)_2_D3 was administered to *Cyp24a1* KO rats, neither 23,25,26(OH)_3_D3 nor 25(OH)D3-26,23-lactone was detected (Fig. 6*C*). These results strongly suggested that CYP24A1 is responsible for the conversion of 25(OH)D3 into 23*S*,25,26(OH)_3_D3 *via* 23*S*,25(OH)_2_D3 (Fig. 7). However, when 23*S*,25*R*,26(OH)_3_D3 was administered to *Cyp24a1* KO rats, 25(OH)D3-26,23-lactone was detected in the plasma (Fig. 6, *B* and *D*). These results strongly suggested that conversion of 25(OH)D3 to 23*S*,25*R*,26(OH)_3_D3 *via* 23*S*,25(OH)_2_D3 is catalyzed by CYP24A1, but enzymes other than CYP24A1 are involved in the conversion of 23*S*,25*R*,26(OH)_3_D3 to 25(OH)D3-26,23-lactone.

**Figure 8:**
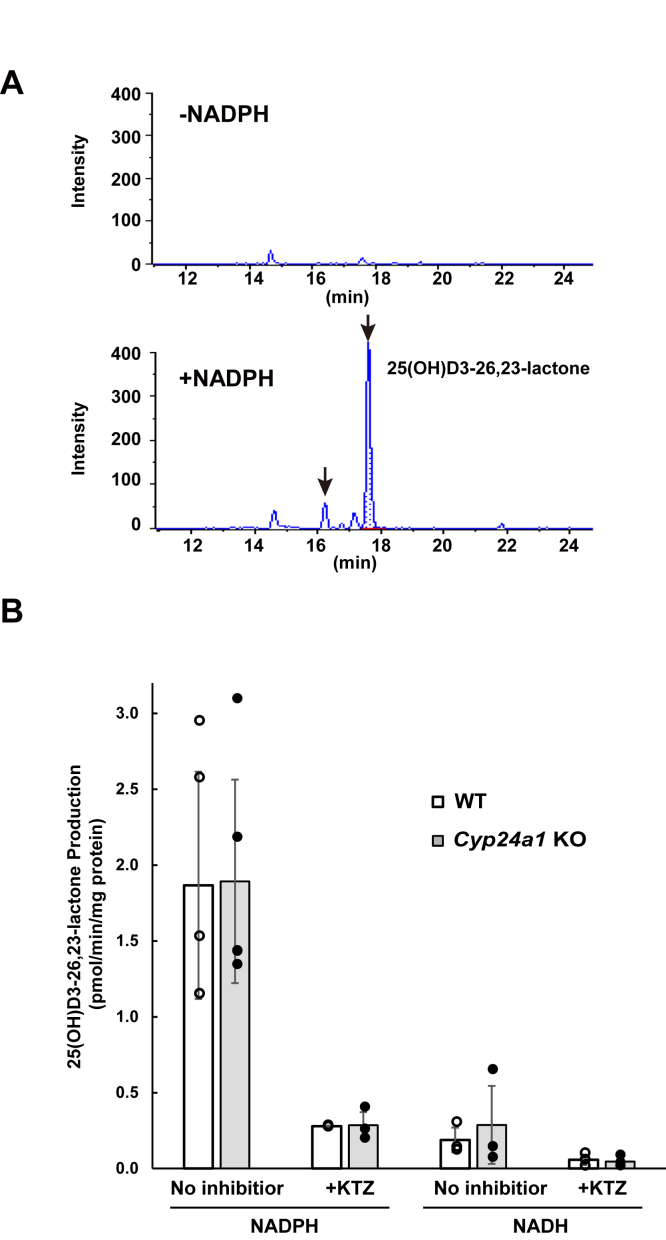
**25(OH)D3-26,23-lactone formation from 23*S*,25*R*,26(OH)**_**3**_**D3 in the liver microsomal fractions prepared from WT or *Cyp24a1* KO rats.***A*, indicates MRM chromatogram to detect -25(OH)D3-26,23-lactone as the metabolites of 23*S*,25*R*,26(OH)_3_D3 in the liver microsomal fractions prepared from *Cyp24a1* KO rats without (*upper*) or with (*bottom*) NADPH. *B*, indicates the inhibitory effects by 1 μM of ketoconazole (KTZ), which is a specific CYP3A inhibitor, on 25(OH)D3-26,23-lactone production from 23*S*,25*R*,26(OH)_3_D3 in the liver microsomal fractions using NADPH or NADH as a coenzyme. *White* and *gray bars* indicate the averages of the individual data of WT (n = 3–4, biological replicate) and *Cyp24a1* KO group (n = 3–4, biological replicates), respectively. Error bars represent standard deviation.

**Figure 9:**
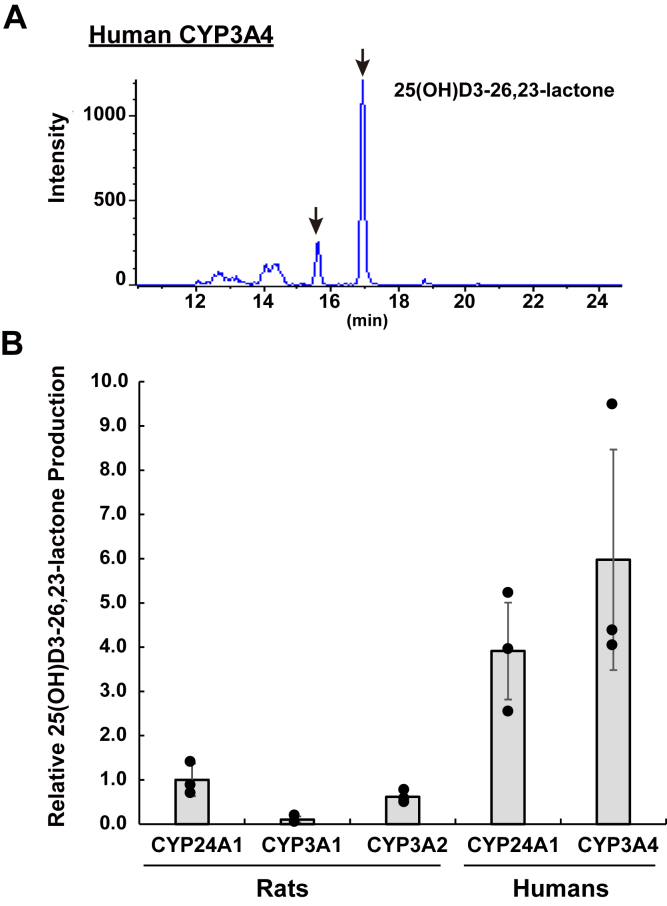
**25(OH)D3-26,23-lactone formation from 23*S*,25*R*,26(OH)**_**3**_**D3 in the recombinant each P450 isoform**. *A*, indicates MRM chromatogram to detect 25(OH)D3-26,23-lactone as the metabolites 23 *S*,25*R*,26(OH)_3_D3 in the recombinant human CYP3A4. *B*, indicates the comparison of the 23*S*,25*R*,26(OH)_3_D3 production by between recombinant CYP24A1 and CYP3A. Each bar indicates the average of the individual data (n = 3; technical replicates). Error bars represent standard deviation.

To identify the enzyme(s) involved in the conversion of 23*S*,25*R*,26(OH)_3_D3 to 25(OH)D3-26,23-lactone, we prepared mitochondrial, microsomal, and cytosolic fractions from *Cyp24a1* KO rat liver and added 23*S*,25*R*,26(OH)_3_D3 to each fraction as a substrate to investigate 25(OH)D3-26,23-lactone formation. Subsequently, 25(OH)D3-26,23-lactone was detected in hepatic microsomal fractions when incubated in the presence of NADPH or NADH (Fig. 8). As reported by Matsunaga *et al*. (24), CYP3A species show NADH-dependent monooxygenase activity. To the best of our knowledge, hepatic microsomal CYPs other than CYP3A show no NADH-dependent activity. A small quantity of 25(OH)D3-26,23-lactone was also detected in the liver mitochondrial fraction in the presence of NADPH or NADH. On the contrary, 25(OH)D3-26,23-lactone was not detected in the cytosolic fraction, even in the presence of NADPH and NADH. These results suggested that enzymes present in the liver microsomal fraction are involved in 25(OH)D3-26,23-lactone formation from 23*S*,25*R*,26(OH)_3_D3. Inhibition studies on 25(OH)D3-26,23-lactone formation revealed that ketoconazole, which is a specific inhibitor of CYP3A, strongly inhibited enzymic activity (Fig. 8*B*). In addition, recombinant rat CYP3A1 and 3A2, as well as human CYP3A4, catalyzed 25(OH)D3-26,23-lactone formation from 23*S*,25*R*,26(OH)_3_D3 (Fig. 9). Inhibitory effect of ketoconazole was also observed in the mitochondrial fraction. The activity of NADPH-cyt.c reductase, which is a marker enzyme of microsomes, was also observed in the mitochondrial fraction (data not shown). These results strongly suggest that the lactone formation detected in the mitochondrial fraction was due to contamination by the microsomal fraction. It was noted that recombinant human CYP1A1, 1A2, 2A6, 2B6, 2C8, 2C9, 2C18, 2C19, 2D6, and 2E1 exhibited no activity (data not shown). These results suggested that CYP24A1 and CYP3A are involved in 25(OH)D3-26,23-lactone formation from 23*S*,25*R*,26(OH)_3_D3.

The enzymatic activity of rat CYP3A1 and CYP3A2 and human CYP3A4 in conversion of 23*S*,25*R*,26(OH)_3_D3 to 25(OH)D3-26,23-lactone was compared with that of rat or human CYP24A1 (Fig. 9*B*). However, their activities cannot be directly compared because CYP3A is a microsomal P450 enzyme whose electron donor is NADPH-P450 oxidoreductase, and CYP24A1 is a mitochondrial P450 whose electron donor is ADX. Rat CYP24A1 and CYP3A2 showed comparable activity, and human CYP24A1 and CYP3A4 showed comparable activity (Fig. 9*B*). Plasma concentrations of 25(OH)D3-26,23-lactone in WT and *Cyp24a1* KO rats at 6 h after administration of 50 μg/kg of 23*S*,25*R*,26(OH)_3_D3 were 101 ± 45 and 80 ± 31 nM (n = 3), respectively, suggesting a greater contribution by CYP3A than CYP24A1 *in vivo*.

When 25(OH)D3 and ketoconazole to WT rats were simultaneously administered, plasma 25(OH)D3-26,23-lactone level was reduced to 50% compared with that without ketoconazole (Fig. S5*, A* and *D*). When 23S,25R,26(OH)3D3 and ketoconazole were simultaneously administered to WT and CYP24A1 rats, 25(OH)D3-26,23-lactone levels were reduced to 44% and 27% at 6 h after administration, respectively, compared with those without ketoconazole (Fig. S5, *B*, *C*, *E*, and *F*). Because the lactone formation in CYP24A1 KO rats appears to depend on CYP3A1/2, it is possible that the activity of CYP3A1/2 was decreased to 27% by the administered ketoconazole. Based on these results, and when the contribution of CYP3A1/2 and CYP24A1 is X and Y, respectively, the following equations could be represented.

X + Y = 1, and 0.27 × X + Y = 0.44

Thus, X and Y are calculated to be 0.77 and 0.23, respectively. These results indicate that the contribution ratio of CYP3A1/2 is 77%, and that of CYP24A1 is 23%.

### Effects of daily administration of 25(OH)D3 to *Cyp24a1* KO rats

**Figure 10:**
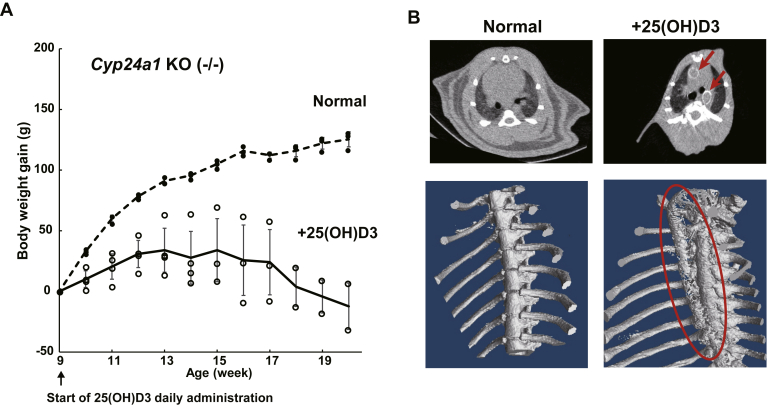
**The effects of daily administration of 25(OH)D3 to *Cyp24a1* KO rats on body weight (*A*) and ectopic calcification (*B*)**. *A*, indicates the body weight comparison between *Cyp24a1* KO rats fed normal (*closed circle*) and 25(OH)D3-containing diet (*open circle*). The *dotted* and *solid lines* indicate the average of normal (n = 3; biological replicates) and 25(OH)D3-containing diet group (n = 4; biological replicates), respectively. Two of four *Cyp24a1* KO rats fed 25(OH)D3-containing diet died before 20 weeks of age. Error bars represent standard deviation. *B*, indicates the μCT image of the chest of *Cyp24a1* KO rats fed normal chow and 25(OH)D3-containing chow. The *red arrow* and *red dotted line* indicate the calcification site in the aorta.

25(OH)D3 was also administered daily to *Cyp24a1* KO rats to clarify the effects of long-term administration. As described in “Materials and Methods,” *Cyp24a1* KO rats were fed normal chow before 9 weeks of age and were then converted to 25(OH)D3-containing chow at a dose of approximately 110 μg/kg body weight/day. Significant weight loss was observed compared with rodents on a normal diet (Fig. 10*A*). Food consumption was also markedly reduced, with initial food consumption of 73.0 ± 12.7 g/kg bw/day (n = 5), which was approximately halved to 36.5 ± 14.6 g/kg bw/day after 6 weeks of 25(OH)D3 treatment. Furthermore, ectopic calcification was observed in *Cyp24a1* KO rats receiving 25(OH)D3-containing chow, based on μCT images. Figure 10*B* illustrates the calcification of the aorta in the chest. On the contrary, when WT rats were fed the same 25(OH)D3-containing diet for 6 weeks, body weight increased as anticipated, with no reduction in food consumption or noticeable phenotypic changes.

Plasma Ca concentrations in the *Cyp24a1* KO rats receiving 25(OH)D3-containing chow for 0, 1, and 2 weeks were 8.8 ± 1.4, 10.1 ± 1.3, and 11.0 ± 0.9 mg/dl, respectively. Thus, plasma Ca levels increased after daily administration of 25(OH)D3, but remained at approximately 11 mg/dl thereafter. Some *Cyp24a1* KO rats died after daily 25(OH)D3 administration for 14–17 weeks. Although this study was conducted at a dose of only approximately 110 μg/kg body weight/day, the effects of long-term administration of 25(OH)D3 on CYP24A1 dysfunction should be investigated in greater detail in the future.

## Discussion

In *in vitro* systems containing recombinant rat CYP24A1 expression system, 25(OH)D3 metabolites in the C-24 pathway were predominant compared with those in the C-23 pathway, and metabolites in the C-23 pathway, except for 23*S*,25(OH)_2_D3, were not detected. However, the end product of the C-23 pathway, 25(OH)D3-26,23-lactone, has been detected as a major metabolite in the plasma of WT rats administered with 25(OH)D3. Although this metabolite has also been reported to be present in humans at elevated 25(OH)D3 levels, the plasma 25(OH)D3-26,23-lactone level was lower than that in mice (16). This appeared to be inconsistent with the fact that compared with rat and mouse CYP24A1, human CYP24A1 displayed higher metabolism in the C-23 pathway (5). Thus, these discrepancies in *in vitro* and *in vivo* 25(OH)D3-26,23-lactone formation between rats, mice, and humans still remain unexplained.

In this study, we revealed that CYP3As are involved in the conversion of 23,25,26(OH)_3_D3 into 25(OH)D3-26,23-lactone, whereas CYP24A1 is responsible for the conversion of 25(OH)D3 into 23,25,26(OH)_3_D3 *via* 23,25(OH)_2_D3 (Fig. 7). Comparison of the amount of 25(OH)D3-26,23-lactone produced by administration of 23*S*,25*R*,26(OH)_3_D3 between WT and *Cyp24a1* KO rats suggests that the contribution of CYP3A1/2 to this reaction could be larger than that of CYP24A1 in WT rats. Enzymatic studies using recombinant human CYP species and the inhibitory effects of ketoconazole strongly suggest that CYP3A plays an essential role in the conversion of 23,25,26(OH)_3_D3 into 25(OH)D3-26,23-lactone in both rats and humans. It is noted that human CYP3A4 is the most important CYP species among human drug-metabolizing P450 species belonging to cytochrome P450 families 1, 2, and 3. Although CYP3A4 catalyzes 4β, 23*R*, and 24*S*-hydroxylation of 25(OH)D3 and 1,25(OH)_2_D3, it has not been given much importance with regard to vitamin D metabolism. However, we revealed another role of CYP3A4 in vitamin D metabolism in this study. Thus, from the viewpoint of drug/drug interaction, CYP3A4-dependent drug/vitamin D interaction may be important. Recently, novel rickets type III has been discovered, which results from the Ile301Thr missense mutation in CYP3A4, causing a 10-fold increase in activity over the normal form (25). The levels of 25(OH)D3 and 1,25(OH)_2_D3 in the plasma of rickets type III patients are severely reduced. Taken together with our results, it might be possible that a high 25(OH)D3-26,23-lactone level could be observed in the plasma of these patients. 25(OH)D3-26,23-lactone and 1,25(OH)_2_D3-26,23-lactone are known to have antagonistic effects on VDRs (18, 26). Thus, low plasma levels of 25(OH)D3 and 1,25(OH)_2_D3 and possibly high plasma levels of 25(OH)D3-26,23-lactone and 1,25(OH)_2_D3-26,23-lactone might additively inhibit VDR-mediated regulation of gene expression to cause rickets type III.

We conducted a metabolic study using *Cyp24a1* KO rats because the body size of these rats was about ten times larger than that of mice, and enough blood could be collected periodically over 48 h. This allowed a detailed analysis of the pharmacokinetics of the various metabolites, as shown in Figure 8 and Table 3. Such analyses appear to be difficult in mice, even if the analyses are sensitive. It is noted that comparisons between WT and *Cyp24a1* KO rats have resolved previous inexplicable issues on 23,26-lactone-25(OH)D3 formation in WT rats.

Recently, SULT2A1 and UGT1A4 have been reported as metabolic enzymes of 25(OH)D3 and 1,25(OH)D3, respectively, in addition to CYP3A (27, 28). In particular, the plasma concentration of sulfate conjugates formed by SULT2A1 was high. The plasma concentrations of 25(OH)D3, 25(OH)D3-3-*O*-sulfate, 24*R*,25(OH)_2_D3, and 4β,25(OH)_2_D3 in healthy humans were 131, 96, 12, and 0.26 nM, respectively (27). Furthermore, some studies demonstrated that the increased expression of VDR induced the transcription of CYP3A (29) and SULT2A1 (30). Based on these results, it is likely that CYP3A and SULT2A1 play critical roles in vitamin D metabolism, especially in cases of CYP24A1 dysfunction. The plasma concentration of sulfate conjugates was not examined in this study, but we will investigate it in more detail in the future.

In humans, CYP24A1 dysfunction sometimes causes elevated plasma levels of 1,25(OH)_2_D3 and is associated with idiopathic infantile hypercalcemia or kidney stones. However, Pronicka *et al.* (31) reported that the plasma 1,25(OH)_2_D3 levels are almost normal in many cases of human CYP24A1 defects. In this study, *Cyp24a1* KO rats showed normal concentrations of 1,25(OH)_2_D3, and its concentration was slightly lower than those in WT rats. It has been reported that 1,25(OH)_2_D3 concentration in the plasma of *Cyp24a1* KO mice is slightly lower than that of *Cyp24a1*-hetero mice (32), which is consistent with our present study. In the case of mice, it has been reported that 50% of *Cyp24a1* KO homogeneous pups with *Cyp24a1* KO parents died before the age of 3 weeks and displayed impaired bone mineralization (32). Since *Cyp24a1* and *Vdr* double-knockout mice normalized bone formation without impairing bone mineralization, St-Arnaud *et al.* (32) speculated that a rapid increase in plasma 1,25(OH)_2_D3 during late gestation in female *Cyp24a1* KO mice, resulting in hypercalcemia, affects calcification during development. In the present study, however, *Cyp24a1* KO rats showed no abnormal phenotype and pups that had *Cyp24a1* KO parents grew normally. At present, the reason for this difference between mice and rats is unclear. Although single 25(OH)D3 administration to *Cyp24a1* KO rats did not cause toxic effects, long-term administration of 25(OH)D3 to the *Cyp24a1* KO rats at 110 μg/kg b.w./day showed significant weight loss and ectopic calcification. In contrast, no side effects were observed in WT rats under the same conditions (Fig. 10). Thus, *Cyp24a1* KO rats treated with vitamin D, 25(OH)D3, or 1,25(OH)_2_D3 could be used as models of renal diseases originating from CYP24A1 dysfunction, although further studies are needed.

In this study, we performed detailed metabolic analysis of 25(OH)D3 *in vivo* by using *Cyp24a1* KO rats generated by genome editing. We conducted a metabolic analysis of natural 25(OH)D3 and concluded that the *Cyp24a1* KO rats could be useful in developing various vitamin D derivatives as pharmaceuticals in the future.

## Experimental procedures

### Materials and chemicals

25(OH)D_3_ was kindly provided by DSM. D6-25-Hydroxyvitamin D3 (26,26,26,27,27,27-D6) (d_6_-25(OH)D3) was purchased from Sigma-Aldrich Co, LLC. Recombinant human or rat drug-metabolizing P450 isoforms were purchased from Corning. Recombinant human or rat CYP24A1 was prepared as described in our previous study (5, 6). HPLC-grade organic solvents were purchased from Kanto chemical CO, Inc. 23*S*,25(OH)_2_D3 and 23*S*,25*R*,26(OH)_3_D3 were synthesized as described in our study (33). 23*S*,25*R*-25(OH)_3_D3-26,23-lactone synthetic standard was kindly given by Dr Keiko Yamamoto (Showa Pharmaceutical University, Tokyo, Japan). Other chemicals were commercially available and of the highest quality.

### Animals and diets

Jcl:Wistar rats were obtained from CLEA Japan Inc. Embryonic microinjection for genome editing was performed by KAC Co, Ltd. The generated *Cyp24a1* KO rats were kept at room temperature (22–26 °C) and in 50–55% humidity with a 12 h light/dark cycle. They were allowed food and water ad libitum and fed F-2 formula diet (Oriental Yeast Co) containing 0.74 w/w% calcium and 2000 IU vitamin D3/kg diet.

All experimental protocols using animals were performed in accordance with the Guidelines for Animal Experiments at Toyama Prefectural University and were approved by the Animal Research and Ethics Committee of Toyama Prefectural University.

### Generation of *Cyp24a1* KO rats and validation of off-target

*Cyp24a1* KO rats were generated by CRISPR/Cas9 genome editing system as well as our previous study (17). The target site was selected to delete the cysteine at position 462 in Exon 10, which is the fifth ligand of heme iron and an active center of CYP24A1 (Fig. S2).

Potential off-target sites (OTS) of CYP24A1 in rat genomes were searched by the CRISPR Direct tool (http://crispr.dbcls.jp/) to valid the off-target events. The OTS region was amplified, purified, and then directly sequenced (Fig. S3).

### Founder generation

Pups delivered from the transferred embryos were obtained by caesarean section on the day of birth and nursed by foster mothers. After weaning, tail tips were biopsied for mutation analysis. Crude genomic DNA was extracted from the tail tips by Lyppo (Wako Pure Chemicals). PCR was performed with KOD Fx Neo (TOYOBO) using the set of primer 5′- ATGAGGAGAATCAGTGGTCCTCCTGGCTGCC-3′, 5′- AGGACTTTTACCCAGCAGAGAGCCAGGTGG-3′. PCR products were purified using FastGene Gel/PCR Extraction Kit (Nippon Gene) according to the manufacture's protocol and directly sequenced and analyzed on an Applied Biosystems 3500 DNA sequencer (Thermo Fisher Scientific) using the BigDye Terminator v3.1 Cycle Sequencing Kit (Thermo Fisher Scientific).

### Measurement of plasma 25(OH)D3 and its metabolites concentration by LC/MS/MS analysis

Plasma concentrations of 25(OH)D3 and its metabolites were measured by using a modified method of LC-APCI-MS/MS (17). Briefly, internal standard d_6_-25(OH)D3 (0.5 ng/10 μl) was added to plasma (40 μl) and precipitated with acetonitrile (200 μl). The supernatant was evaporated and the residue was extracted with ethyl acetate (400 μl) and distilled water (200 μl). Extracted 25(OH)D3 and its metabolites in plasma were derivatized by 4-[2-(6,7-dimethoxy-4-methyl-3-oxo-3,4-dihydroquinoxalyl)ethyl]-1,2,4-triazoline-3,5-dione (DMEQ-TAD) and analyzed by LC/MS/MS using the selection of the specific pair of m/z values associated to precursor and product ion (Supple Table 1) with an MS/MS multiple reaction monitoring (MRM) method. Calibration curves were obtained by representing the peak area ratio between each concentration of analyte and internal standard (d_6_-25(OH)D3). Concentrations of each metabolite, except 25(OH)D3, were calculated considering the ratio of extraction efficiency between 25(OH)D3 and each metabolite.

### Measurement of plasma 1,25(OH)_2_D3 with ELISA kit

Plasma concentration of 1,25(OH)_2_D3 was measured using 1,25-(OH)_2_ Vitamin D ELISA Kit (Immundiagnostik) as described previously (11). Prior to the assay, solid-phase extraction using Chromabond XTR (Immundiagnostik) and Sep-pak Silica Cartridge (Waters) was performed according to the manufacture's protocol.

### Measurement of plasma Ca, phosphorus (P), and parathyroid hormone (PTH) concentrations

The plasma Ca and P concentrations were measured using the Calcium E-Test Wako and Phospha C-Test Wako (FUJIFILM Wako Pure Chemical Corporation), respectively. The plasma PTH concentration was determined using the Rat Intact PTH ELISA Kit (Immutopics Inc).

### Phenotype of femura

Micro CT-scans analysis of femura was performed using an X-ray CT system (Latheta LCT-200; Hitachi Aloka Medical). Parameters used for the CT scans were same as described in our previous study (17). Von Kossa staining and Toluidine Blue staining were performed as described in our previous study (17).

### Administration of a single dose of 25(OH)D3, 23*S*,25(OH)_2_D3, or 23*S*,25*R*,26(OH)_3_D3 to WT or *Cyp24a1* KO rats

Certain amount of 25(OH)D3, 23*S*,25(OH)_2_D3, or 23*S*,25*R*,26(OH)_3_D3 was suspended to 300 μl of corn oil, and single dose of each vitamin D3 was orally administered to WT or *Cyp24a1* KO rats at the dose of 200 μg/kg-body weight for 25(OH)D3 or 50 μg/kg-body weight for 23*S*,25(OH)_2_D3 and 23*S*,25*R*,26(OH)_3_D3. Blood samples were collected from the jugular vein at 0, 2, 4, 6, 8, 12, 24, 48 h after administration of each vitamin D3. All of the blood samples were immediately centrifuged at 1000*g* for 10 min, and the resultant supernatant (plasma) was stored at –80 °C until analysis.

In order to investigate the effect of CYP3A inhibition *in vivo*, 30 mg/kg-body weight of ketoconazole was coadministrated with each of 25(OH)D3 or 23*S*,25*R*,26(OH)_3_D3, and the blood samples were collected at 6 h after administration.

### Daily administration of 25(OH)D3 to *Cyp24a1* KO rats

25(OH)D3 was also daily dietary administrated to *Cyp24a1* KO rats to clarify effects of long-term administration. 25(OH)D3 containing food was prepared as described in our previous study (17). Briefly, pellet diet containing 1.5 mg 25(OH)D3 per 1 kg F-2 normal diet was prepared by Oriental Yeast Co. Normal F-2 diet was fed prior to 9-week age, and after that 25(OH)D3-containing diet was fed. The average daily food intake was 20.0 ± 5.0 g in the beginning of dietary 25(OH)D3 administration, and the food intake per kg body weight was 73.0 ± 12.7 g/kg bw/day (N = 5). Thus, the dose of the 25(OH)D3 was calculated to be 110 ± 19 μg/kg bw/day in the beginning of dietary 25(OH)D3 administration.

### Preparation of liver mitochondrial and microsomal fractions and analysis of productivity of 23,26-lactone-25(OH)D3 from 23*S*,25*R*,26(OH)_3_D3 in each fraction

The liver mitochondrial and microsomal fractions were prepared from WT and *Cyp24a1* KO rats, and the productivity of 25(OH)D3-23,26-lactone in each mitochondrial and microsomal fraction was analyzed by modified methods described in the previous study (11). The mitochondrial fraction was sonicated on ice for 1 min in 10 s bursts with 1 min of cooling. It was then incubated in 100 mM Tris-HCl buffer (pH 7.4) containing 5000 nM ADX, 500 nM ADR, 10 μM 23*S*,25*R*,26(OH)_3_D3, and 1 mM NADPH or NADH at 37 °C for 30 min. The microsomal fraction was incubated in 100 mM phosphate buffer (pH 7.4), 10 μM 23*S*,25(OH)_2_D3 or 23*S*,25*R*,26(OH)_3_D3, and 1 mM NADPH or NADH at 37 °C for 30 min. The substrate and its metabolites were extracted and analyzed as described in the section, Measurement of plasma 25(OH)D3 and its metabolites concentration by LC/MS/MS analysis.

### Productivity of 23,26-lactone-25(OH)D3 from 23*S*,25*R*,26(OH)_3_D3 by recombinant CYP24A1, rat CYP3A1/2, and human CYP3A4

Metabolism of 23*S*,25*R*,26(OH)3D3 by human and rat CYP24A1 was examined with the same methods as described in our previous study (34). Briefly, The reaction mixture containing 100 mM Tris-HCl (pH 7.5), 1 mM EDTA, 2 μM ADX, 200 nM ADR, 20 nM recombinant CYP24A1, 10 μM 23*S*,25*R*,26(OH)_3_D3, and 1 mM NADPH was incubated for 30 min. Metabolism of 23*S*,25*R*,26(OH)_3_D3 by human CYP3A4 or rat 3A1 and 3A2 was examined as followed. The reaction mixture containing 100 mM phosphate buffer (pH 7.5), 20 nM recombinant P450, 10 μM 23*S*,25*R*,26(OH)_3_D3, and 1 mM NADPH was incubated for 30 min. Reaction was stopped by addition of ethyl acetate, and metabolites were extracted and analyzed as described in the section, Measurement of plasma 25(OH)D3 and its metabolites concentration by LC/MS/MS analysis.

### Statistical analysis

Student's *t*-test was used to identify significant differences between WT and *Cyp24a1* KO rats (*p* < 0.05).

## Data availability

All data generated or analyzed in the present study are included in this article or the data repositories listed in References.

## Supporting information

This article contains supporting information (17).

## Conflict of interest

The authors declare that they have no conflicts of interest with the contents of this article.

## References

[1] Jones G., Prosser D.E., Kaufmann M. (2012). 25-Hydroxyvitamin D-24-hydroxylase (CYP24A1): Its important role in the degradation of vitamin D. Arch. Biochem. Biophys..

[2] Sakaki T., Kagawa N., Yamamoto K., Inouye K. (2005). Metabolism of vitamin D3 by cytochromes P450. Front. Biosci..

[3] Nesterova G., Malicdan M.C., Yasuda K., Sakaki T., Vilboux T., Ciccone C., Horst R., Huang Y., Golas G., Introne W., Huizing M., Adams D., Boerkoel C.F., Collins M.T., Gahl W.A. (2013). 1,25-(OH)2D-24 hydroxylase (CYP24A1) deficiency as a cause of nephrolithiasis. Clin. J. Am. Soc. Nephrol..

[4] Schlingmann K.P., Kaufmann M., Weber S., Irwin A., Goos C., John U., Misselwitz J., Klaus G., Kuwertz-Broking E., Fehrenbach H., Wingen A.M., Guran T., Hoenderop J.G., Bindels R.J., Prosser D.E. (2011). Mutations in CYP24A1 and idiopathic infantile hypercalcemia. N. Engl. J. Med..

[5] Sakaki T., Sawada N., Komai K., Shiozawa S., Yamada S., Yamamoto K., Ohyama Y., Inouye K. (2000). Dual metabolic pathway of 25-hydroxyvitamin D3 catalyzed by human CYP24. Eur. J. Biochem..

[6] Kusudo T., Sakaki T., Abe D., Fujishima T., Kittaka A., Takayama H., Hatakeyama S., Ohta M., Inouye K. (2004). Metabolism of A-ring diastereomers of 1α,25-dihydroxyvitamin D3 by CYP24A1. Biochem. Biophys. Res. Commun..

[7] Pedersen J.I., Hagenfeldt Y., Bjorkhem I. (1988). Assay and properties of 25-hydroxyvitamin D3 23-hydroxylase. Evidence that 23,25-dihydroxyvitamin D3 is a major metabolite in 1,25-dihydroxyvitamin D3-treated or fasted Guinea pigs. Biochem. J..

[8] Prosser D.E., Kaufmann M., O'Leary B., Byford V., Jones G. (2007). Single A326G mutation converts human CYP24A1 from 25-OH-D3-24-hydroxylase into -23-hydroxylase, generating 1α,25-(OH)2D3-26,23-lactone. Proc. Natl. Acad. Sci. U. S. A..

[9] Napoli J.L., Pramanik B.C., Partridge J.J., Uskokovic M.R., Horst R.L. (1982). 23S,25-dihydroxyvitamin D3 as a circulating metabolite of vitamin D3. Its role in 25-hydroxyvitamin D3-26,23-lactone biosynthesis. J. Biol. Chem..

[10] Tanaka Y., Wichmann J.K., Paaren H.E., Schnoes H.K., DeLuca H.F. (1980). Role of kidney tissue in the production of 25-hydroxyvitamin D3-26,23-lactone and 1α,25-dihydroxyvitamin D3-26,23-lactone. Proc. Natl. Acad. Sci. U. S. A..

[11] Nishikawa M., Yasuda K., Takamatsu M., Abe K., Nakagawa K., Tsugawa N., Hirota Y., Tanaka K., Yamashita S., Ikushiro S., Suda T., Okano T., Sakaki T. (2019). Generation of 1,25-dihydroxyvitamin D3 in Cyp27b1 knockout mice by treatment with 25-hydroxyvitamin D3 rescued their rachitic phenotypes. J. Steroid Biochem. Mol. Biol..

[12] Masuda S., Byford V., Arabian A., Sakai Y., Demay M.B., St-Arnaud R., Jones G. (2005). Altered pharmacokinetics of 1α,25-dihydroxyvitamin D3 and 25-hydroxyvitamin D3 in the blood and tissues of the 25-hydroxyvitamin D-24-hydroxylase (Cyp24a1) null mouse. Endocrinology.

[13] Kaufmann M., Martineau C., Arabian A., Traynor M., St-Arnaud R., Jones G. (2019). Calcioic acid: *In vivo* detection and quantification of the terminal C24-oxidation product of 25-hydroxyvitamin D3 and related intermediates in serum of mice treated with 24,25-dihydroxyvitamin D3. J. Steroid Biochem. Mol. Biol..

[14] Kaufmann M., Lee S.M., Pike J.W., Jones G. (2015). A high-calcium and phosphate rescue diet and VDR-expressing transgenes normalize serum vitamin D metabolite profiles and renal Cyp27b1 and Cyp24a1 expression in VDR null mice. Endocrinology.

[15] Kaufmann M., Morse N., Molloy B.J., Cooper D.P., Schlingmann K.P., Molin A., Kottler M.L., Gallagher J.C., Armas L., Jones G. (2017). Improved screening test for idiopathic infantile hypercalcemia confirms residual levels of serum 24,25-(OH)2 D3 in affected patients. J. Bone Miner. Res..

[16] Kaufmann M., Gallagher J.C., Peacock M., Schlingmann K.P., Konrad M., DeLuca H.F., Sigueiro R., Lopez B., Mourino A., Maestro M., St-Arnaud R., Finkelstein J.S., Cooper D.P., Jones G. (2014). Clinical utility of simultaneous quantitation of 25-hydroxyvitamin D and 24,25-dihydroxyvitamin D by LC-MS/MS involving derivatization with DMEQ-TAD. J. Clin. Endocrinol. Metab..

[17] Nishikawa M., Yasuda K., Takamatsu M., Abe K., Okamoto K., Horibe K., Mano H., Nakagawa K., Tsugawa N., Hirota Y., Horie T., Hinoi E., Okano T., Ikushiro S., Sakaki T. (2020). Generation of novel genetically modified rats to reveal the molecular mechanisms of vitamin D actions. Sci. Rep..

[18] Ishizuka S., Ishimoto S., Norman A.W. (1982). Biological activity assessment of 25-hydroxyvitamin D3-26,23-lactone in the rat. FEBS Lett..

[19] Ishizuka S., Yamaguchi H., Yamada S., Nakayama K., Takayama H. (1981). Stereochemistry of 25-hydroxyvitamin D3-26,23-lactone and 1α, 25-dihydroxyvitamin D3-26,23-lactone in rat serum. FEBS Lett..

[20] Higashi T., Awada D., Shimada K. (2001). Simultaneous determination of 25-hydroxyvitamin D2 and 25-hydroxyvitamin D3 in human plasma by liquid chromatography-tandem mass spectrometry employing derivatization with a Cookson-type reagent. Biol. Pharm. Bull..

[21] St-Arnaud R. (1999). Targeted inactivation of vitamin D hydroxylases in mice. Bone.

[22] Bouillon R., Okamura W.H., Norman A.W. (1995). Structure-function relationships in the vitamin D endocrine system. Endocr. Rev..

[23] Ishizuka S., Norman A.W. (1987). Metabolic pathways from 1α,25-dihydroxyvitamin D3 to 1α,25-dihydroxyvitamin D3-26,23-lactone. Stereo-retained and stereo-selective lactonization. J. Biol. Chem..

[24] Matsunaga T., Higuchi S., Watanabe K., Kageyama T., Ohmori S., Yamamoto I. (2005). Effective NADH-dependent oxidation of 7beta-hydroxy-delta8-tetrahydrocannabinol to the corresponding ketone by Japanese monkey hepatic microsomes. Biol. Pharm. Bull..

[25] Roizen J.D., Li D., O'Lear L., Javaid M.K., Shaw N.J., Ebeling P.R., Nguyen H.H., Rodda C.P., Thummel K.E., Thacher T.D., Hakonarson H., Levine M.A. (2018). CYP3A4 mutation causes vitamin D-dependent rickets type 3. J. Clin. Invest..

[26] Ishizuka S., Norman A.W. (1986). The difference of biological activity among four diastereoisomers of 1α,25-dihydroxycholecalciferol-26,23-lactone. J. Steroid Biochem..

[27] Wong T., Wang Z., Chapron B.D., Suzuki M., Claw K.G., Gao C., Foti R.S., Prasad B., Chapron A., Calamia J., Chaudhry A., Schuetz E.G., Horst R.L., Mao Q., de Boer I.H. (2018). Polymorphic human sulfotransferase 2A1 mediates the formation of 25-hydroxyvitamin D3-3-O-sulfate, a major circulating vitamin D metabolite in humans. Drug Metab. Dispos..

[28] Wang Z., Wong T., Hashizume T., Dickmann L.Z., Scian M., Koszewski N.J., Goff J.P., Horst R.L., Chaudhry A.S., Schuetz E.G., Thummel K.E. (2014). Human UGT1A4 and UGT1A3 conjugate 25-hydroxyvitamin D3: Metabolite structure, kinetics, inducibility, and interindividual variability. Endocrinology.

[29] Qin X., Wang X. (2019). Role of vitamin D receptor in the regulation of CYP3A gene expression. Acta Pharm. Sin. B.

[30] Chatterjee B., Echchgadda I., Song C.S. (2005). Vitamin D receptor regulation of the steroid/bile acid sulfotransferase SULT2A1. Methods Enzymol..

[31] Pronicka E., Ciara E., Halat P., Janiec A., Wojcik M., Rowinska E., Rokicki D., Pludowski P., Wojciechowska E., Wierzbicka A., Ksiazyk J.B., Jacoszek A., Konrad M., Schlingmann K.P., Litwin M. (2017). Biallelic mutations in CYP24A1 or SLC34A1 as a cause of infantile idiopathic hypercalcemia (IIH) with vitamin D hypersensitivity: Molecular study of 11 historical IIH cases. J. Appl. Genet..

[32] St-Arnaud R., Arabian A., Travers R., Barletta F., Raval-Pandya M., Chapin K., Depovere J., Mathieu C., Christakos S., Demay M.B., Glorieux F.H. (2000). Deficient mineralization of intramembranous bone in vitamin D-24-hydroxylase-ablated mice is due to elevated 1,25-dihydroxyvitamin D and not to the absence of 24,25-dihydroxyvitamin D. Endocrinology.

[33] Kawagoe F., Sugiyama T., Yasuda K., Uesugi M., Sakaki T., Kittaka A. (2019). Concise synthesis of 23-hydroxylated vitamin D3 metabolites. J. Steroid Biochem. Mol. Biol..

[34] Yasuda K., Tohyama E., Takano M., Kittaka A., Ohta M., Ikushiro S., Sakaki T. (2018). Metabolism of 2α-[2-(tetrazol-2-yl)ethyl]-1α,25-dihydroxyvitamin D3 by CYP24A1 and biological activity of its 24*R*-hydroxylated metabolite. J. Steroid Biochem. Mol. Biol..

[35] Tieu E.W., Tang E.K., Tuckey R.C. (2014). Kinetic analysis of human CYP24A1 metabolism of vitamin D via the C24-oxidation pathway. FEBS J..

