# Supplementary material for: Elucidation of metabolic pathways of 25-hydroxyvitamin D3 mediated by CYP24A1 and CYP3A using Cyp24a1 knockout rats generated by CRISPR/Cas9 system
Source: J Biol Chem. 2021 Apr 15;296:100668. doi: 10.1016/j.jbc.2021.100668 (PMC8134072; doi:10.1016/j.jbc.2021.100668)
Supplement: Figures S1 to S5 and Table S1 [file mmc1.pdf]

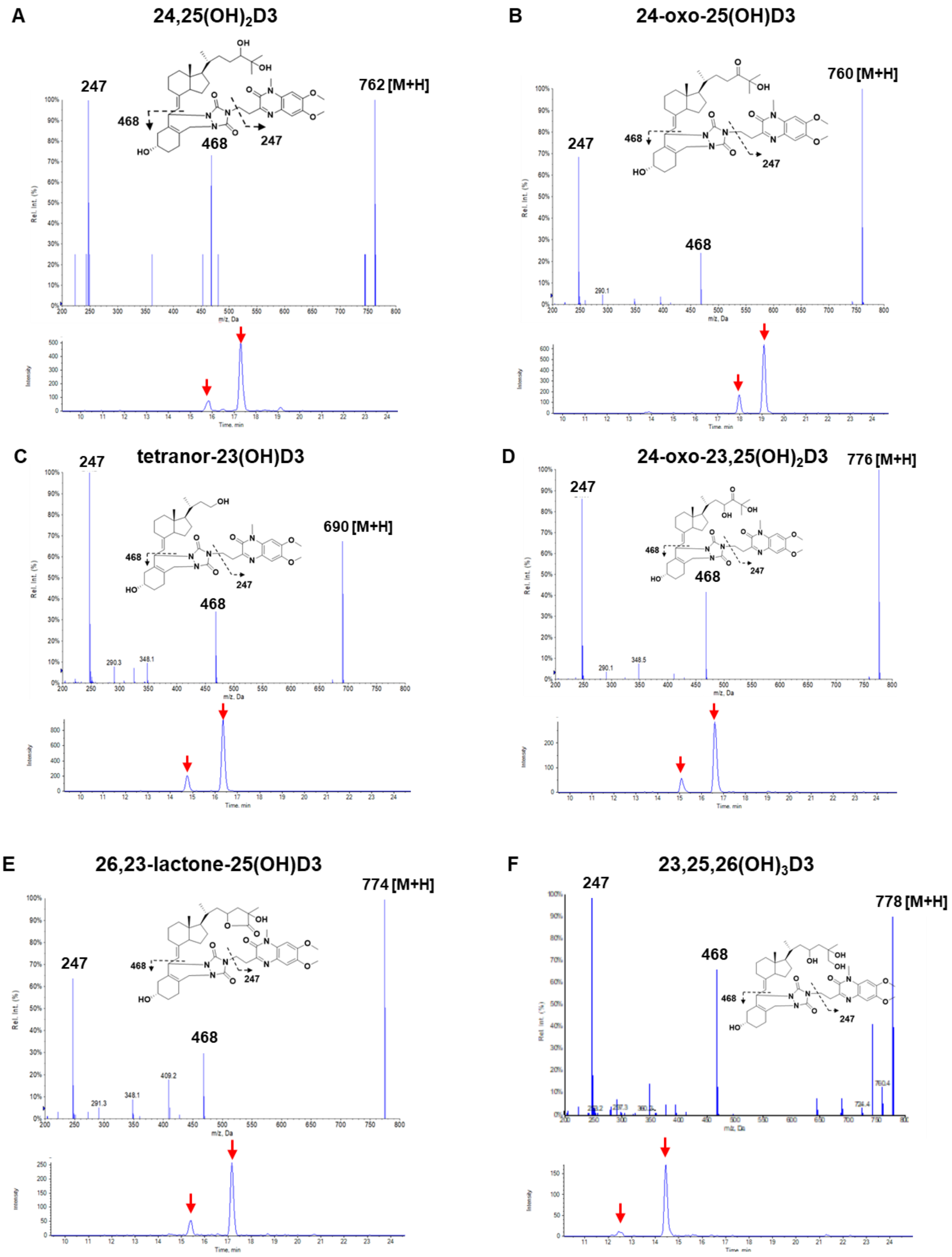

Supplemental Fig.1.

MRM charts and MS fragment of parent ion of 24,25$(OH)_2$D3 (A), 24-oxo-25(OH)D3 (B), tetranor-23(OH)D3 (C), 24-oxo-23,25$(OH)_2$D3 (D), 25(OH)D3-23,26-lactone (E), and 23,25,26$(OH)_3$D3 (F)

a

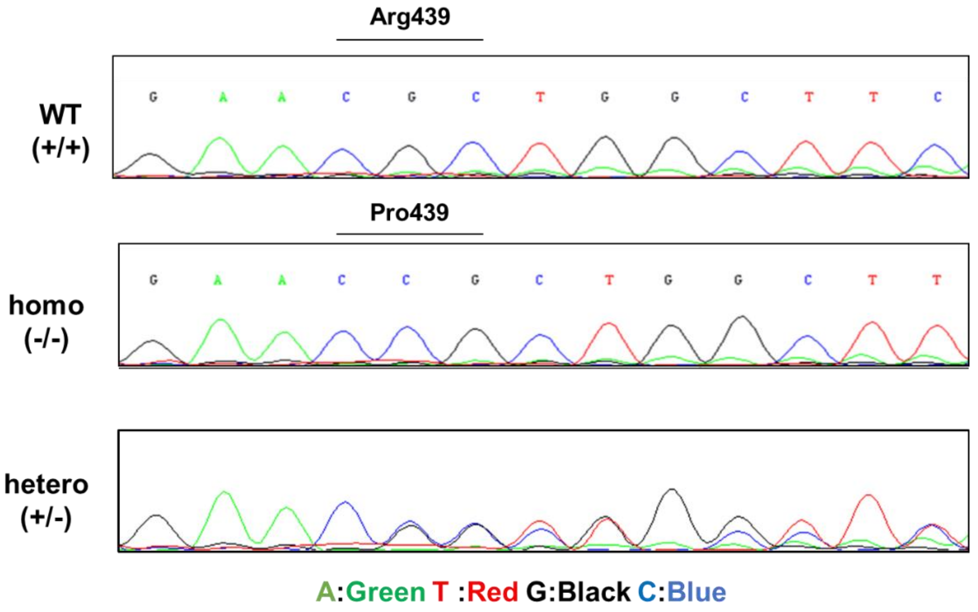

b

```
WT           1  MSCPIDKRRTLIAFLRRLRDLGQPPRSVTSKASASRAPKEVPLCPLMTDGETRNVTSLPG  60
CYP24A1 KO   1  MSCPIDKRRTLIAFLRRLRDLGQPPRSVTSKASASRAPKEVPLCPLMTDGETRNVTSLPG  60

WT          61  PTNWPLLGSLLEIFWKGGLKKQHDTLAEYHKKYGQIFRMKLGSFDSVHLGSPSLLEALYR  120
CYP24A1 KO  61  PTNWPLLGSLLEIFWKGGLKKQHDTLAEYHKKYGQIFRMKLGSFDSVHLGSPSLLEALYR  120

WT         121  TESAHPQRLEIKPWKAYRDHRNEAYGLMILEGQEWQRVRSAFQKKLMKPVEIMKLDKKIN  180
CYP24A1 KO 121  TESAHPQRLEIKPWKAYRDHRNEAYGLMILEGQEWQRVRSAFQKKLMKPVEIMKLDKKIN  180

WT         181  EVLADFLERMDELCDERGRIPDLYSELNKWSFESICLVLYEKRFGLLQKETEEEALTFIT  240
CYP24A1 KO 181  EVLADFLERMDELCDERGRIPDLYSELNKWSFESICLVLYEKRFGLLQKETEEEALTFIT  240

WT         241  AIKTMMSTFGKMMVTPVELHKRLNTKVWQAHTLAWDTIFKSVKPCIDNRLQRYSQQPGAD  300
CYP24A1 KO 241  AIKTMMSTFGKMMVTPVELHKRLNTKVWQAHTLAWDTIFKSVKPCIDNRLQRYSQQPGAD  300

WT         301  FLCDIYQQDHLSKKELYAAVTELQLAAVETTANSLMWILYNLSRNPQAQRRLLQEVQSVL  360
CYP24A1 KO 301  FLCDIYQQDHLSKKELYAAVTELQLAAVETTANSLMWILYNLSRNPQAQRRLLQEVQSVL  360

WT         361  PDNQTPRAEDLRNMPYLKACLKESMRLTPSVPFTTRTLDKPTVLGEYALPKGTVLTLNTQ  420
CYP24A1 KO 361  PDNQTPRAEDLRNMPYLKACLKESMRLTPSVPFTTRTLDKPTVLGEYALPKGTVLTLNTQ  420

                                                    ▼
WT         421  VLGSSEDNFEDSHKFRPERWLQKEKKINPFAHLPFGIGKRMCIGRRLAELQLHLALCWII  480
CYP24A1 KO 421  VLGSSEDNFEDSHKFRPEPLASKGEKDQPLRSSPIRHREENVHRAPAG*             469

WT         481  QKYDIVATDNEPVEMLHLGILVPSRELPIAFRPR*                           515
```

Supplemental Fig. 2

Nucleotide sequence profiles of the WT (+/+) and *Cyp24a1* KO (-/-) homo and hetero

(+/-) rats (a) and the comparison of amino acid sequence of WT (+/+) and *Cyp24a1* KO (-/-) homo (b). Black arrow shows the cysteine residue at position 462, which is the fifth ligand of heme iron and the active center.

a

Target sequence :
ATAAGTTCAGACCCGAACGCTGG
database Rat genome, RGSC 6.0/rn6 (Jul, 2014)
query AGACCCGAACGCNGG
count 1

OTS No.	Chromosome	Strand	Start	End	Forward Primer (5'-3')	Reverse Primer (5'-3')
1	chr9	+	94487751	94487765	TCCTCCTTTGGCAGATTTCCCC	GGTGCCTTTGTGGCGAAGATTG

b OTS1: ATAAGTTCAGACCCGAACGCTGG

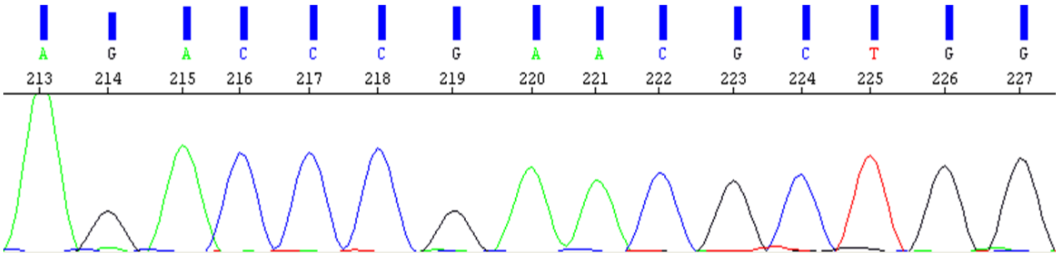

Supplemental Fig. 3

Potential OTS of *Cyp24a1* target site and primer sequence for OTS analysis (a) and its sequence result (b). The forward or reverse primer was used for direct sequencing. No OTS events occurred in potential OTS.

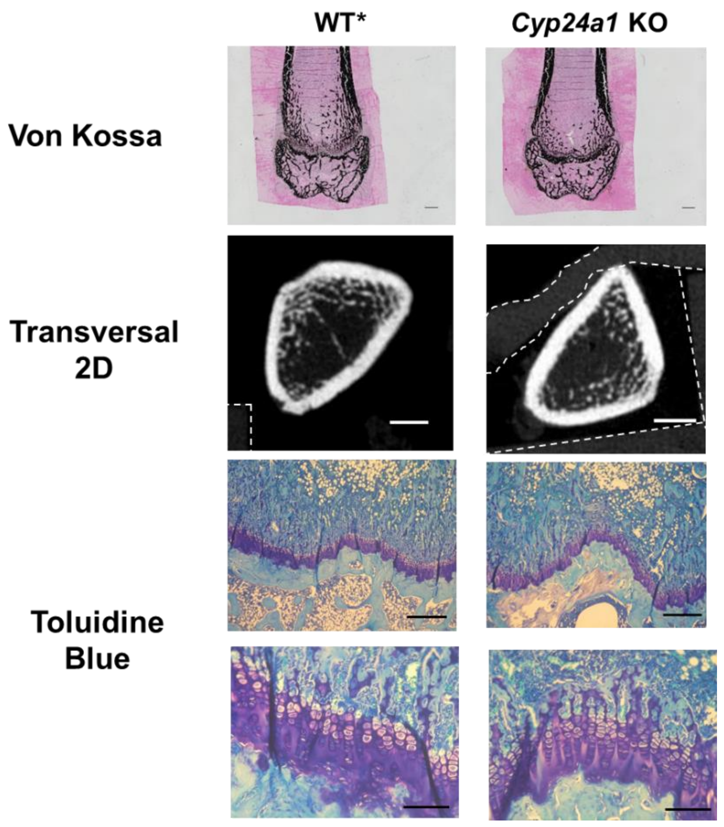

* Previous our study (Ref.17)

Supplemental Fig. 4

Phenotypes of femora of WT (left) and *Cyp24a1* KO (right) rats. Top panels, von Kossa staining of distal femora (Scale bar: 1000 μm); second panels, 2D μ-CT images of horizontal distal femur sections (Scale bar: 1000 μm); third and bottom panels, toluidine blue staining of epiphyseal cartilages (Scale bar: 500 and 100 μm for third and bottom panels, respectively) were performed. There were no significant differences between WT and *Cyp24a1* KO rats. Broken lines in 2D μ-CT images; sample holder signal.

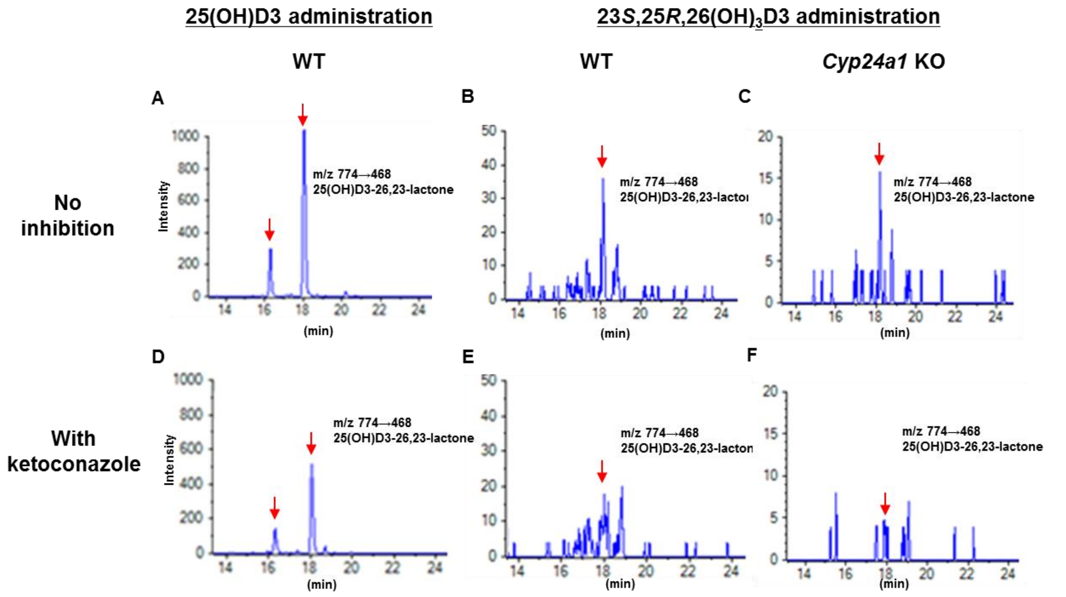

Supplemental Fig. 5

MRM chromatogram of DMEQ-adducts of 25(OH)D3-23,26-lactone in the plasma of WT (A, B, D, and E) and *Cyp24a1* KO (C and F) rats 6hr after single administration of 200 μg/kg of 25(OH)D3 (A and D) or 50 μg/kg of 23*S*,25*R*,26(OH)$_3$D3 (B, C, E and F) with (D-F) or without (A-C) ketoconazole. Arrows indicate the peaks derived 25(OH)D3-23,26-lactone.

Supplemental Table 1

MRM precursor/product ion transitions in determination of DMEQ-TAD adducts of 25(OH)D3 and its metabolites.

	precursor ion m/z	product ion m/z
25(OH)D3	746.4	468.1
24,25$(OH)_2$D3	762.4	468.1
24-oxo-25(OH)D3	760.4	468.1
24-oxo-23,25$(OH)_2$D3	776.4	468.1
tetranor-23(OH)D3	690.4	468.1
25(OH)D3-26,23-lactone	774.4	468.1
23,25,26$(OH)_3$D3	778.4	468.1
d6-25(OH)D3	752.4	468.1
